# An overlooked mechanism underlying the attenuated temperature response of soil heterotrophic respiration

**DOI:** 10.1098/rsif.2022.0276

**Published:** 2022-07-20

**Authors:** Xiaoxian Zhang, Peter A. Whalley, Andrew S. Gregory, W. Richard Whalley, Kevin Coleman, Andrew L. Neal, Sacha J. Mooney, Kenichi Soga, Tissa H. Illangasekare

**Affiliations:** ^1^ Sustainable Soils and Crops, Rothamsted Research, Harpenden AL5 2JQ, UK; ^2^ School of Mathematics, University of Edinburgh, Peter Guthrie Tait Road, Edinburgh EH9 3FD, UK; ^3^ Net Zero and Resilient Farming, Rothamsted Research, North Wyke EX20 2SB, UK; ^4^ School of Biosciences, University of Nottingham, Sutton Bonington Campus, Loughborough, Leicestershire LE12 5RD, UK; ^5^ Department of Civil and Environmental Engineering, University of California–Berkeley, Berkeley, CA 94720, USA; ^6^ Centre for Experimental Study of Subsurface Environmental Processes, Colorado School of Mines, Golden, CO, USA

**Keywords:** soil respiration, oxygen dissolution and diffusion, temperature response of soil respiration, microscopic soil structure

## Abstract

Biogeochemical reactions occurring in soil pore space underpin gaseous emissions measured at macroscopic scales but are difficult to quantify due to their complexity and heterogeneity. We develop a volumetric-average method to calculate aerobic respiration rates analytically from soil with microscopic soil structure represented explicitly. Soil water content in the model is the result of the volumetric-average of the microscopic processes, and it is nonlinearly coupled with temperature and other factors. Since many biogeochemical reactions are driven by oxygen (O_2_) which must overcome various resistances before reaching reactive microsites from the atmosphere, the volumetric-average results in negative feedback between temperature and soil respiration, with the magnitude of the feedback increasing with soil water content and substrate quality. Comparisons with various experiments show the model reproduces the variation of carbon dioxide emission from soils under different water content and temperature gradients, indicating that it captures the key microscopic processes underpinning soil respiration. We show that alongside thermal microbial adaptation, substrate heterogeneity and microbial turnover and carbon use efficiency, O_2_ dissolution and diffusion in water associated with soil pore space is another key explanation for the attenuated temperature response of soil respiration and should be considered in developing soil organic carbon models.

## 1. Introduction

Soil contains more organic carbon (1580 billion t C) than plant biomass (610 billion t C) and atmospheric carbon dioxide (CO_2_) (750 billion t C) combined. As a consequence, small shifts in soil organic carbon (SOC) could have significant consequences for global warming [1]. An understanding of the mechanisms underlying SOC dynamics and representing them in SOC models adequately is crucial to predicting the outcomes of climate warming and land management practices upon SOC stocks [2]. However, a thorough understanding is challenging due to the complexities of biogeochemical reactions involved in SOC cycling [3].

Of all biotic and abiotic factors, temperature and water have the greatest influence upon biogeochemical processes in soil [4]. Although their roles in homogeneous media are fairly well understood, controversy arises when applying this understanding to soils because of their complexity and heterogeneity [5]. Microbial activity in soil is patchy and biogeochemical reactions proceed only where substrates and enzymes exist [6]. Soil variables measured from sampling without information on substrate accessibility can thus give rise to erroneous conclusions when used to calculate biogeochemical reactions [7]. One example is the observed reduced temperature response of aerobic microbial respiration, where the underlying mechanisms have been a point of contention for decades [8–11]. Changes in microbial physiology with temperature and substrate heterogeneity have both been postulated, but there is little consensus about their relative significance [3].

Previous studies on the temperature response of soil respiration have focused on biological processes; overlooking the fact that biogeochemical reactions are driven by bioavailability of oxygen (O_2_) and substrate accessibility within the pore space. Resistance against O_2_ dissolution in soil water and its subsequent movement limits its delivery to microbes, evident as the pervasiveness of anaerobicity in relatively dry soils [12]. While soil water was thought to keep microbes hydrated and carry dissolved substrates and enzymes away and towards microsites [13], experiments suggest that reduced substrate availability due to soil water decrease is far more important than dehydration [14]. Within soil pore space, gaseous O_2_ first dissolves at the water–air interface before diffusing to aerobic microbes (reactive sites). Respiration generates localized O_2_ concentration gradients between the water–air interface and reactive sites. These gradients vary with temperature and soil water content, as rising temperature reduces O_2_ dissolution while a change in soil water content reshapes the water–air interface and the distance between the sites of O_2_ dissolution and microsites. As a result, the influences of temperature and soil water content on microbial activity have been postulated to be coupled: a change in one factor is likely to alter the response of soil respiration to the other. However, most SOC models decouple the effects of temperature and soil water content using separate moisture and temperature functions to describe their respective influence [15,16]. There are no systematic studies of the potential errors associated with this approach; indirect evidence indicates that the temperature coefficient, *Q*_10_ (or activation energy, *E_a_*) which characterizes the temperature sensitivity of soil respiration, varies with soil moisture [17]. When predicting the response of soil respiration to global warming, a small change in *Q*_10_ can lead to substantial differences [18].

The temperature function used in most SOC models is *Q*_10_ or the Arrhenius kinetic model [4]. By contrast, the moisture functions employed are diverse, including both empirical formulae and mechanistic models [15]. While the empirical functions are phenomenological, most mechanistic models are based on the influence of soil water on the diffusion of gaseous O_2_ and aqueous substrates. This approach overlooks the fact that biogeochemical reactions alter local O_2_ concentration gradients and hence the diffusion of dissolved O_2_. For soil, these models predict a fixed optimal moisture content where respiration is maximized. This is inconsistent with experimental results which show that the optimal soil water content for soil respiration varies with temperature [19–22], microbial activity [23] and even soil depth [24]. This implies that the effect of moisture on respiration is modulated by temperature [25], and that their combined influence is more complicated than described by the separated moisture and temperature functions used in current models [3].

As well as mediating O_2_ dissolution and diffusion, soil water content also controls microbial access to substrates [13,26,27]. The consensus view arising from decades of study is that increasing soil water content facilitates substrate movement, increasing microbial access [4,28]. Some models account for this by introducing a moisture-dependent barrier between substrates and reactive sites [22,29]. While this is rational for substrates and enzymes, it does not apply to O_2_ because O_2_ must dissolve at water–air interface before becoming bioavailable for respiring microbes. Increasing water content of a dried soil initially increases O_2_ dissolution, but once a threshold is reached, increasing soil water content further reduces O_2_ supply because of the reduced area of water–air interface across which O_2_ must dissolve, and the increased distance for dissolved O_2_ to travel to reactive sites [30]. Dissolution of O_2_ and its diffusion control biogeochemical reactions in soil [31], but they are difficult to model due to their complexity [32]. Consequently, most SOC models do not consider O_2_ explicitly, probably based on an erroneous perception that O_2_ in the topsoil is not a limiting factor [33]. Decades of studies have demonstrated anaerobic reactions persist even when soil is relatively dry, especially in the rhizosphere [31,34,35].

Given the importance of soil architecture in modulating water distribution and O_2_ dissolution and diffusion, as well as the influence that biogeochemical reactions impose on local O_2_ concentration gradients between water–air interface and reactive sites, we hypothesized that the reduced O_2_ dissolution and increased microbial metabolism at raised temperatures attenuate the temperature response of soil respiration. We developed a volumetric-average method to incorporate microscale soil architecture to calculate soil respiration. Considering that soil architecture and its associated microscopic processes are complicated, to make the calculation analytically tractable, we adopted several rational simplifications. These include (i) gaseous O_2_ concentration in a soil sample is uniform as O_2_ diffuses four orders of magnitude faster in air than in liquid water [36]; (ii) biogeochemical reactions in a soil sample are in a quasi-steady state where the amount of O_2_ dissolved at water–air interfaces is the same as the amount of O_2_ reduced by aerobically respiring microbes, and that the mass of O_2_ respired is the same as the mass of O_2_ diffusing from water–air interfaces to all reactive sites; (iii) O_2_ reduction by aerobic microbial activity at hydrated reactive sites in a soil sample is proportional to the dissolved O_2_ concentration at reactive sites; (iv) the majority of soil microbes adopt a ‘waiting’ strategy to acquire substrates and O_2_ [37]. Volumetrically averaging microscopic processes over the hydrated pore space in a soil sample yields an analytical model to calculate respiration; soil water content in the model is the result of the volumetric-average and nonlinearly coupled with temperature and other factors. Such coupling has been conjectured since the 1970s [38,39]: here we demonstrate its existence and use theory to infer that a change in one factor reshapes the response of soil respiration to the other.

## 2. Material and methods

### 2.1. Theoretical analysis

**Figure 1.**
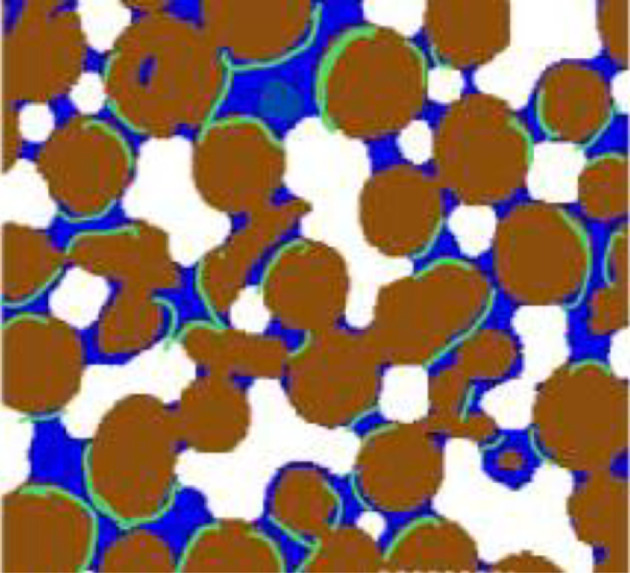
Schematic of microscopic processes controlling aerobic microbial respiration at the pore scale in soil. Brown represents the soil matrix, white regions represent air and blue represents water. Gaseous O_2_ (red) dissolves at the water–air interface; dissolved O_2_ (yellow) moves to aerobic microbes (green) adjacent to wetted pore walls where O_2_ is respired.

Figure 1 depicts the microscopic processes considered in the model. Gaseous O_2_ first dissolves at the water–air interface before moving in water as dissolved O_2_; soil organic matter associated with the matrix is decomposed enzymatically by exoenzymes. At hydrated sites with the coexistence of substrates, aerobic microbes take up dissolved O_2_ and substrates. The movement of dissolved O_2_ and substrates are confined to the regions enclosed by the water–air interface and wetted pore walls. It is described by the following equations, with dissolved organic carbon and O_2_ as the limiting substrates [4,40]:

2.1 $$\begin{array}{rl} \frac{\partial c_{O}}{\partial t}= & \nabla D\nabla c_{o}-v_{\max}N\frac{c_{D}}{k_{D}+c_{D}}\frac{c_{o}}{k_{o}+c_{o}}\\ \mathrm{and}\;\frac{\partial c_{D}}{\partial t}= & \nabla D_{D}\nabla c_{D}-(1-\beta )v_{\max}N\frac{c_{D}}{k_{D}+c_{D}}\frac{c_{o}}{k_{o}+c_{o}}+s_{DOC}. \end{array}\}$$

Variable nomenclature is given in appendix A. O_2_ enters the system via dissolution at the water–air interface, which is described by a first-order kinetic process [41]

2.2 $$d_{o}=\alpha (C_{eq}-c_{0}),$$

where *d_o_* is O_2_ dissolution rate over a unit water–air interfacial area, *α* is the dissolution rate coefficient, *c*_0_ is the dissolved O_2_ concentration at the water–air interface and *C*_eq_ is the saturated dissolved O_2_ concentration, calculated from the Henry's law [42].

Compared with the timeframe over which soil water content changes, pore-scale processes are rapid and at a quasi-steady state over daily temporal scale. The aim of this paper is not to solve equation (2.1), but to develop a method to volumetrically average it throughout a soil sample to calculate the total reaction rate. To make the calculation analytically tractable while maintaining the key processes illustrated in figure 1, we made some rational simplifications as explained above. For a soil water content of *θ*, the total area of wetted pore walls in a soil is represented by *A_ws_*(*θ*), and the spatial variation of the wetted pore wall in the Cartesian coordinate system is described by function s(x,y,z). If the number of aerobic microbes on the wetted wall located at (x,y,z) is *n*(*s*), the total aerobic microbes on hydrated microsites in the soil is ${∯}_{A_{ws}}n(s)\cdot ds$, which reduces to *A_ws_*(*θ*) · *n*_0_ if the microbes are uniformly distributed in that *n*(*s*) = *n*_0_. Similarly, the total water–air interfacial area is represented by *A_wa_*(*θ*), and the spatial variation of the water–air interface in the Cartesian coordinate system is described by function *s′*(*x*, *y*, *z*). If the dissolved O_2_ concentration at the water–air interface located at (*x*, *y*, *z*) is c_0_(*s′*), the O_2_ dissolution rate in the soil is ${∯}_{A_{wa}}\alpha [C_{eq}-c_{0}(s')]\cdot ds'$. The Michaelis–Menten constant associated with O_2_ in equation (2.1) regulates microbial growth, varying with microbial species and substrate quality [43,44]. Since we consider whole microbial communities, it is approximated by $Kc_{o}/(k_{o}+c_{o})\approx \kappa c_{o}$ [33].

At steady state, the rate of microbial reduction of O_2_ in a soil sample is balanced by the O_2_ dissolution rate at water–air interfaces. When soil water content is *θ*, we have

2.3 $${∯}_{A_{wa}}\alpha \left[C_{eq}-c_{0}(s')\right]ds'={∯}_{A_{ws}}n(s)\left(1-\beta \right)v_{\max}\kappa c_{o}\frac{c_{D}}{k_{D}+c_{D}}ds.$$

The dissolved O_2_ concentration at the water–air interface in a soil sample is approximately constant as the gaseous O_2_ concentration is constant. By contrast, substrate concentrations and the number of aerobically respiring microbes vary over the wetted pore wall. Equation (2.3) is hence calculated by

2.4 $$\alpha A_{wa}\left(C_{eq}-c_{0}\right)=n'\left(1-\beta \right)v_{\max}\frac{C_{D}}{k_{D}+C_{D}}\kappa {∯}_{A_{ws}}c_{o}ds,$$

where *C_D_* and *n*′ are the average substrate concentration and the average number of aerobic microbes over a unit area of wetted pore walls in the soil, respectively, when soil water content is *θ*. The diffusion of O_2_ in water is slow and its concentration over the wetted pore walls, *c*_o_, varies. The integral in equation (2.4) is approximated by

2.5 $${∯}_{A_{ws}}c_{o}ds=A_{ws}C_{o},$$

where *C_o_* is the average dissolved O_2_ concentration over the wetted pore wall. We thus have

2.6 $$\alpha A_{wa}(C_{\mathrm{eq}}-c_{0})=n^{\prime }(1-\beta )v_{\max}\kappa \frac{C_{D}}{k_{D}+C_{D}}A_{ws}C_{o},$$

Diffusion of O_2_ from the water–air interface to reactive sites depends on their spatial separation and local concentration gradients. If the average hydraulic distance between air–water interfaces and wetted pore walls is *L*, and the average cross-sectional area in soil water for O_2_ to diffuse from water–air interface to the microsites is *A_D_*, the overall diffusive flux is

2.7 $$Q=D\frac{c_{0}-C_{o}}{L}A_{D}.$$

Approximating *A_D_* by the mean of the water–air interfacial areas and wetted pore wall areas, i.e. $A_{D}=0.5(A_{ws}+A_{wa})$, at steady state, the mass balance requires that the diffusive flux *Q* equates the total O_2_ respiration rate, i.e.

2.8 $$D\frac{c_{0}-C_{o}}{L}\frac{A_{ws}+A_{wa}}{2}=n^{\prime }(1-\beta )\;v_{\max}\kappa A_{ws}\frac{C_{D}}{k_{D}+C_{D}}C_{o}.$$

Solving for *c*_0_ yields:

2.9 $$\begin{array}{rl} c_{0} & =\left(1+k\frac{2L\cdot A_{ws}}{D(A_{ws}+A_{wa})}\right)C_{o}\\ \;\mathrm{and}\;k & =n^{\prime }(1-\beta )v_{\max}\kappa \frac{C_{D}}{k_{D}+C_{D}}. \end{array}\}$$

Substituting equation (2.9) into equation (2.6) gives

2.10 $$C_{o}=\frac{\alpha A_{wa}}{\alpha A_{wa}\;\left[1+2L\cdot (k/D)\cdot A_{ws}/(A_{ws}+A_{wa})\right]\;+kA_{ws}}C_{\mathrm{eq}}.$$

The total respiration rate from the soil can be calculated as follows:

2.11 $$\begin{array}{rl} \Omega =\; & k\cdot A_{ws}\cdot C_{\mathrm{eq}}\cdot E_{r}\\ \;\mathrm{and}\;E_{r}= & {\left[1+\frac{k}{\alpha }\left(\frac{2\alpha }{D}\frac{L\cdot A_{ws}}{\left(A_{ws}+A_{wa}\right)}+\frac{A_{ws}}{A_{wa}}\right)\right]}^{-1} \end{array}\},$$

where *E_r_* < 1 is a feedback factor emerging from the volumetric-average, describing the reduction in respiration due to O_2_ dissolution and diffusion in soil water. Physically, *k* represents the potential demand of aerobic microbes over a unit area of wetted pore wall for O_2_ when O_2_ is not a limiting factor. It depends on temperature, substrate quality/quantity, the number of microbes and their metabolic rates. The hydraulic distance between the water–air interface and the microsites increases with soil water content, approximated by [41]

2.12 $$L=\lambda \frac{A_{ws}}{A_{wa}},$$

where *λ* is constant depending upon soil architecture.

The effect of O_2_ dissolution and diffusion on soil respiration is described by the O_2_ dissolution rate coefficient and the molecular diffusion coefficient of dissolved O_2_, respectively. When O_2_ dissolution is significantly faster than microbial respiration, i.e. k/α≪1, the feedback factor reduces to

2.13 $$E_{r}={\left[1+\frac{2k\lambda }{D}\frac{A_{ws}}{\left(A_{ws}+A_{wa}\right)}\frac{A_{ws}}{A_{wa}}\right]}^{-1},$$

with O_2_ diffusion being the limiting factor. When dissolved O_2_ diffusion is significantly faster than microbial respiration, i.e. kL/D≪1, the feedback factor reduces to

2.14 $$E_{r}={\left[1+\frac{k}{\alpha }\frac{A_{ws}}{A_{wa}}\right]}^{-1},$$

with O_2_ dissolution being the limiting factor.

The influence of pore geometry and water distribution within the pore space is described by a combination of the water–air interfacial area, the wetted pore wall area and the hydraulic distance separating them. Temperature influences soil respiration in two ways. Physiologically, rising temperature increases microbial metabolic rate, described by the Arrhenius kinetic equation [5],

2.15 $$v_{\max}=v\cdot \exp\left[-\frac{E_{a}}{R(T+273)}\right].$$

Physically, increasing temperature reduces O_2_ dissolution but enhances its diffusion. At one atmospheric pressure, changes in saturated concentrations of dissolved O_2_ and its molecular diffusion coefficient with temperature are described by [36],

2.16 $$\begin{array}{rl} & C_{\mathrm{eq}}=0.434\exp(0.000064T^{2}-0.0114T+1.161),\\ & D=0.434\exp[-4.41+773.8/(T+273)-2564.4/{\left(T+273\right)}^{2}]. \end{array}$$

### 2.2. Water–air interfaces and wetted pore walls

As soil water content increases, the wetted pore wall area increases monotonically. However, the water–air interfacial area first increases and then declines. To investigate if such changes can be described by general formulae, we simulated water distribution in over 100 soil samples as described in our previous work [45,46]. For all soil samples, the changes in water–air interfacial area and the wetted pore wall area with soil saturation follow similar trends (electronic supplementary material, figure S1). Normalized by the volume of the soil sample, their changes with soil saturation, ϴ, can be described by

2.17 $$\begin{array}{rl} A_{wa}= & A_{a}{\left(1+\varepsilon -\Theta \right)}^{\sigma }\Theta^{\tau }\\ \;\mathrm{and}\;A_{ws}= & A_{w}\Theta^{\mu }, \end{array}\}$$

where *ɛ* is a parameter to represent that when the soil surface is opened to the atmosphere, its water–air interfacial area is non-zero when the soil is fully saturated; others are soil parameters.

### 2.3. The model

The rate of O_2_ dissolution is much faster than its diffusion in water as commonly assumed in hydrogen fuel cells [41]. Since microbial reduction of O_2_ is slower than electrochemical reduction in fuel cells, all analysis in what follows is based on equation (2.13). For a soil sample, combining equation (2.13) and equation (2.17) gives

2.18 $$\begin{array}{rl} \Omega (\Theta ,\;T) & =k\cdot A_{w}\Theta^{\mu }\cdot C_{\mathrm{eq}}\cdot E_{r},\\ E_{r} & =\frac{1}{1+2\cdot k\cdot \lambda \cdot \Theta^{\mu -\tau }{\left(1+\varepsilon -\Theta \right)}^{-\sigma }[D\cdot A+D\cdot A^{2}\cdot \Theta^{\tau -\mu }{\left(1+\varepsilon -\Theta \right)}^{\sigma }{]}^{-1}},\\ A & =A_{a}/A_{w}\\ \;\mathrm{and}\;k & =n^{\prime }(1-\beta )\cdot \kappa \cdot v\cdot \exp\left(-\frac{E_{a}}{R(T+273)}\right)\frac{C_{D}}{k_{D}+C_{D}}. \end{array}\}$$

### 2.4. Implementation

Equation (2.18) is used to calculate soil respiration rates. The influence of temperature and substrates is described by the parameter *k*. Apart from *k*, all other parameters are related to soil architecture. Mathematically, each parameter in equation (2.18) can take an arbitrary value, but in validating the model against experimental data, we take all soil structure parameters as a set, calculating it by mining a soil image dataset consisting of more than 100 X-ray images we have accumulated over the past decade for soils with various textures ranging from clay loam to sandy soils [45–49]. Using a method we developed previously [50], we calculated liquid water distribution within the pore geometry at different water contents, as well as the associated water–air interfaces and wetted pore walls for each soil image. For each soil image, we substituted its water–air interfacial areas and wetted pore walls into equations (2.17) and (2.18), and then adjusted parameter *k*, which can be either constant or varies with pore size (see the Results and discussion section for details), until the calculated respiration rates from equation (2.18) matched experimental data.

## 3. Results and discussion

### 3.1. Comparison with experimental data

**Figure 2.**
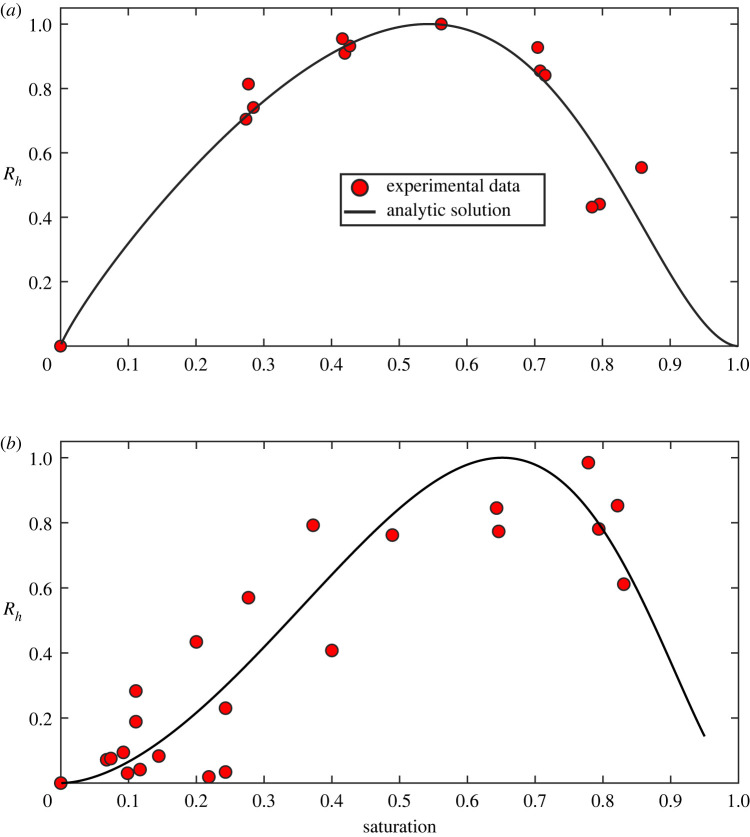
Comparison of experimental data (symbols) and model results (solid lines) using exemplar soil architectures under different soil saturations from an incubation experiment using a well-mixed sandy soil where the potential demand of aerobic microbes for O_2_ was relatively uniform (*a*); for a field experiment using a loamy soil where microbes and substrates are sparser in small pores than in large pores (*b*).

Our first example is an incubation experiment that measured soil respiration rates under different soil saturations using a repacked sandy soil [51]. All respiration rates were normalized by the maximum respiration at the optimal soil water content. Figure 2*a* compares the measured and modelled results using the soil architecture shown in the electronic supplementary material, figure S1A. They agreed well, indicating the model captures the key processes underpinning respiration at different soil saturations.

Our second example is a field experiment designed to investigate the response of soil respiration to changes in soil moisture [52]. Field soil is more heterogeneous than sieved and repacked soil. Its respiration often exhibits the ‘Birch’ phenomenon [53], suggesting that microbes in small pores respire less than those in large pores due to pore-scale variation in substrates and microbial composition [54,55]. We modelled this by allowing *k* to increase with pore size. Since water progressively fills small to large pores as soil water increases, we described this pore-scale substrate and microbial heterogeneity by allowing *k* to increase linearly with soil water content. Figure 2*b* shows the comparison. The model reproduces the change in respiration from dry to wet soil, revealing that O_2_ dissolution and diffusion in soil water regulates the response of aerobic heterotrophic soil respiration to soil water change.

Our final example was chosen to demonstrate that the model captures the nonlinear coupling between soil water and temperature in their effect on respiration using an incubation experiment that involved both moisture and temperature gradients [56]. The influence of soil water content was measured experimentally by maintaining a constant temperature, while the effect of temperature was measured by keeping the soil moisture content constant. We first calculated the soil structure parameters by calibrating the model against respiration rates measured at different saturations at 15°C and then used these parameters and equation (2.15) to predict respiration rate variation when temperature was increased from 5 to 30°C. The molecular diffusion coefficient of dissolved O_2_ and the saturated O_2_ concentration at different temperatures were calculated from equation (2.16). The potential demand of the reactive sites for O_2_ at temperature *T* is *k_T_*, calculated as follows based on its value at 15°C (*T*_15_) calibrated for obtaining the soil structure parameters:

3.1 $$k_{T}=k_{15}\exp\left[\frac{E_{a}}{R}\left(\frac{1}{T_{15}}-\frac{1}{T}\right)\right],$$

where *k*_15_ is the value of *k* at 15°C, and *T*_15_ and *T* are absolute temperature (K).

Moriyama *et al.* [56] estimated the apparent activation energy based on respiration rates measured across a range of temperatures, effectively a bulk estimate representing the collective impact of all factors which influence the temperature response of soil respiration. In our model, *E*_*a*_ is intrinsic, determined by microbial composition and molecular structures of the substrates. However, because substrates and microbial composition vary with pore size [55,57], even the intrinsic *E_a_* itself is by necessity an average value, representing the average of the intrinsic *E_a_* of the substrates and microbes on different hydrated reactive sites. In this example, we considered the average intrinsic *E_a_* as an unknown and calibrated it to obtain the results which matched the measured respiration rates at different temperatures.

**Figure 3.**
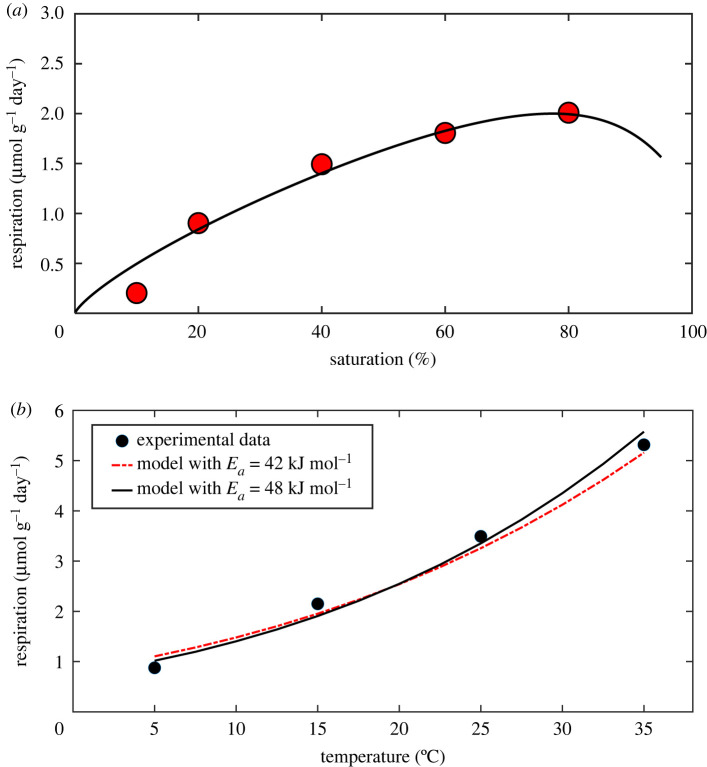
(*a*) Comparison of measured (symbols) and calculated respiration rates (solid line) using calibrated soil structure and potential O_2_ demand when soil saturation is increased from 7 to 80% at 15°C. (*b*) The calibrated model was used to predict respiration rates for temperatures between 5 and 35°C at a constant water saturation of 60%. The increase in microbial metabolic activity with temperature is described by the Arrhenius kinetic model with an intrinsic activation energy (*E_a_*) of 48 kJ mol^−1^. For comparison, we also calculated respiration rates using the bulk *E*_*a*_ of 42 kJ mol^−1^ measured from the experimental data [56].

For comparing respiration rates, we normalized all variables in the model and multiplied the dimensionless respiration rates calculated using these normalized variables by a single scalar to match the experimental data. As an illustration, figure 3*a* compares the measured and calculated respiration rates under different soil water saturations at 15°C; figure 3*b* compares the measured and predicted respiration rates when the soil was 60% saturated as temperature increases from 5 to 35°C using an intrinsic *E_a_* of 48 kJ mol^−1^. They agree well, indicating that the combined influence of soil water and temperature on soil respiration is nonlinear and is captured well by our model. For comparison, we also calculated the temperature response of respiration rates directly using the bulk *E_a_* (45 kJ mol^−1^) reported in Moriyama *et al.* [56] and plot the results in figure 5*b*. The predictions using the intrinsic *E_a_* = 48 kJ mol^−1^ are slightly better. Because of the limited experimental data, in terms of matching the measured data, the difference between the two activation energies is not significant, but it corroborates that intrinsic *E_a_* is higher than bulk *E_a_* [5].

### 3.2. Attenuated temperature response of respiration

Nonlinear coupling of the influence of soil water content and temperature in equation (2.18) indicates that the temperature response of respiration is regulated by soil water content, and the strength of their coupling is modulated by *E_a_*. This differs from previous studies which attributed the temperature response of respiration to microbial physiology and substrate quality [58–65]. Equation (2.18) separates soil structure and its associated physical processes from other factors in their role in mediating the temperature response of soil respiration. This is important but has been overlooked. For example, increasing temperature from 5 to 35°C reduces the saturated O_2_ concentration in water from approximately 14 to 7 mg l^−1^ [36]; ignoring this fall would overestimate the thermal adaptation of soil microbes. The effect of these physical factors is described by the feedback factor (equation (2.16)), whose magnitude varies with soil water content, *E_a_* and temperature (electronic supplementary material, figures S2).

**Figure 4.**
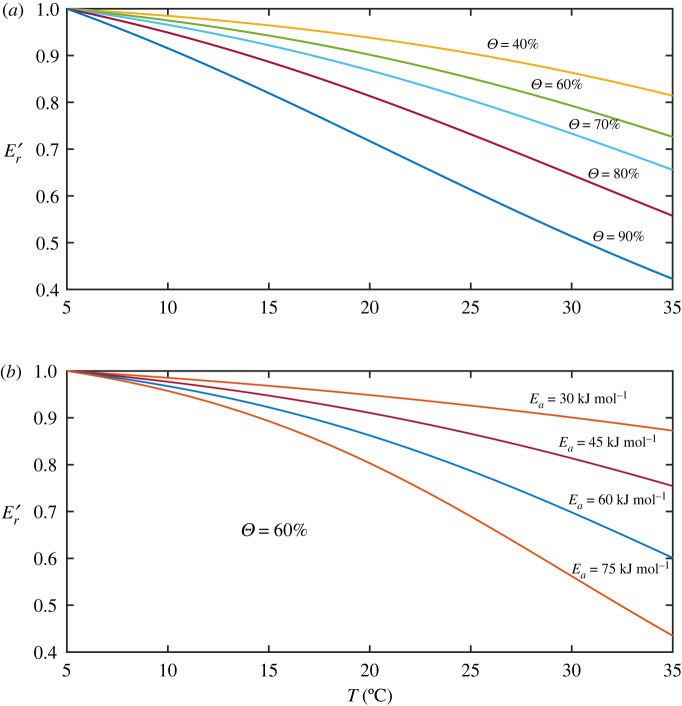
The feedback factor describing the attenuation of respiration rates decreases with the increase in soil saturation (*a*) and activation energy *E_a_* (*b*). $E_{r}^{\prime }$ is the ratio between the feedback factor at temperature *T* to the feedback factor at 5°C.

The attenuated temperature response of soil respiration is regulated by both *E_a_* and soil water content. Figure 4 shows the change in the feedback factor with temperature at different soil saturations (figure 4*a*) and *E*_*a*_ (figure 4*b*), respectively, calculated using soil parameters for the example in figure 3. To allow comparison, the feedback factor calculated for each saturation (or *E_a_*), was normalized by the value of the feedback factor at 5°C. Depending on soil saturation (or *E_a_*), increasing temperature from 5 to 35°C could dampen respiration rates by approximately 60% due to reduced O_2_ dissolution and increased hydraulic resistance against O_2_ flux from the water–air interface to the reactive sites as temperature rises. This is consistent with experimental results that reducing O_2_ supply substantially reduced respiration [52]; it is also corroborated by the results of a whole soil profile experiment where *E_a_* in the subsoil was greater than that in the topsoil [66,67]. While biological factors such as differences in SOC quality and microbial community composition between the top- and sub-soils are also likely to be important [68,69], O_2_ dissolution and diffusion are critical factors because the subsoil is more saturated, and the preferential consumption of O_2_ by roots and microbes in the topsoil limits O_2_ diffusion to reactive sites in the subsoil. Not accounting explicitly for the reduced O_2_ dissolution and diffusion in the subsoil would underestimate the intrinsic temperature sensitivity of soil respiration, giving rise to a reduced *E_a_* or *Q*_10_.

### 3.3. Non-unique optimal soil moisture for aerobic respiration

**Figure 5.**
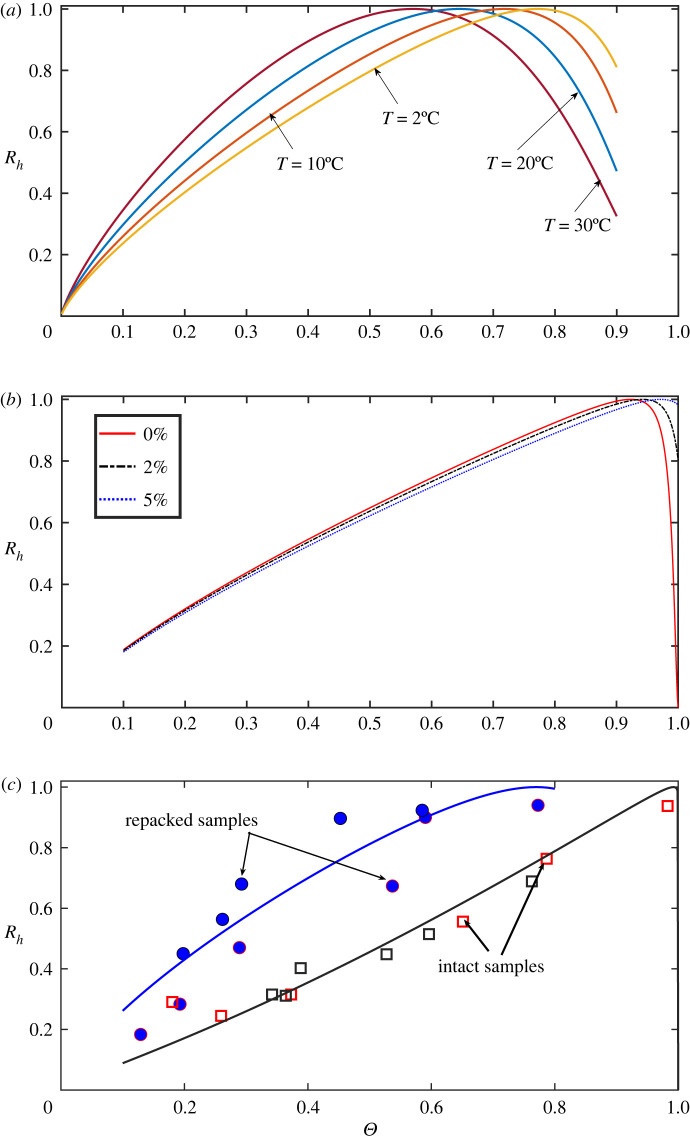
(*a*) The optimal saturation for maximal aerobic heterotrophic respiration (*R*_h_) traditionally used in moisture functions is not unique, but is temperature (*T*) dependent because of its nonlinear coupling with soil moisture. (*b*) Maintaining the soil surface open in incubation experiments alters the response of *R*_*h*_ to soil water when soil is close to saturation; the effects increase from non-open (0%) to having 5% of pores in direct contact with the atmosphere. (*c*) Accounting for microbial and substrate heterogeneities explains the variation of experimentally measured saturation–respiration relationships [71]; open squares are data measured from a field-structured loamy soil; solid circles are data measured after sieving-repacking; solid lines are results calculated from the model.

The analytical model derived from the volumetric-average reveals that the influence of soil water content and temperature on soil respiration is more complicated than described by separate moisture and temperature functions [70]. For each temperature, however, there is still an optimal soil water content at which respiration rate peaks when other factors are fixed. Normalizing respiration rates at different soil water contents by this maximum gives a curve which is equivalent to the moisture function used in most SOC models [15]. Taking the soil parameters calibrated for figure 3 as an example, we calculated the moisture function at different temperatures with other factors kept constant (figure 5*a*). It is evident that for the same soil, the optimal soil saturation varies with temperature. As temperature increases from 2 to 30°C for the example shown in figure 5*a*, the optimal soil saturation decreases, from approximately 80 to 55%; this range covers the soil saturation deemed optimal (60%) for aerobic microbes used in most incubation experiments [9,56,59].

In soils lacking liable carbon substrates, the respiration rate measured in incubation experiments typically increases linearly as soil water increases rather than following the trend shown in figures 2 and 3 [72]. Even when soils are approximately saturated, a significant amount of CO_2_ continues to emit from the soils [56,71]. The likely reason is that the soil surface remains open to the atmosphere. When soil is fully saturated with water, O_2_ continues to dissolve at the soil surface and move into the soil. Thus, depending on the quantity and quality of SOC within the soil and the soil surface areas, the change in respiration with soil water content can be either approximately linear, nonlinear or bell-shaped; all these responses are captured by our model. As an illustrative example, figure 5*b* compares how the soil surface opening affects the response of respiration to soil water content when microbial activity is low and other parameters are the same.

### 3.4. Substrate and microbial heterogeneity

Aerobic respiration measured during short-term experiments exhibits an exponential increase as temperature rises [10,38], while the change in respiration with soil moisture is inconsistent, ranging from a linear increase [73,74], concave increase [51], convex increase [71], to convex increase followed by a plateau before declining [52]. Repacking a sieved soil can also dramatically change the moisture response of respiration compared with undisturbed soil [71]. In the latter, physical constraints are likely to prevent microbes from entering small pores, and there is evidence that substrate quality in small pores is less energetically favourable than those in large pores [57]. These heterogeneities are a possible cause of the broader variation in the moisture response of soil respiration. Representing all these heterogeneities in a single analytical model is a formidable task, but their effects can still be quantitatively accounted for by allowing the density of microbial numbers on wetted pore walls and substrate concentrations to vary with pore size [54]. For example, when soil water content is low, only small pores are filled by water in which microbes are less active as most microbes in larger pores are in dormancy [75]. As soil water content increases and larger pores become progressively refilled by water, dormant microbes become more active and substrate availability increases contemporaneously. Allowing the density of microbes over the wetted pore walls and substrate concentration to increase with pore size in the model can represent these pore-scale heterogeneities to produce a diverse set of saturation–respiration relationships. Figure 5*c* shows how including such pore-scale heterogeneity reshapes the moisture response of respiration rates, in comparison with an experiment which measured respiration from intact and repacked soil cores [71]. We acknowledge that other factors are also likely to play a role in these diverse respiration–saturation relationships as those in figure 5*c*, but here we highlight the importance of microscopic soil architecture and physical processes which are typically overlooked in most data analysis and SOC models. Under certain circumstances, they might overwhelm biotic factors and physiological change in microbes in mediating the moisture and temperature response of microbial activity [13].

## 4. Conclusion

We develop a volumetric-average method, with soil architecture and microscopic physical processes represented explicitly, to calculate aerobic respiration analytically from soil samples. Soil water content in the model is the result of the volumetric-average, and it is nonlinearly coupled with temperature and other factors. Comparison with experimental data shows the model reproduces respiration rates measured from soils with both water content and temperature gradients. Incorporating microbial and substrate heterogeneities into the model can explain the diverse moisture– and temperature–respiration relationships. The model demonstrates that, alongside thermal adaptation, substrate heterogeneity and carbon use efficiency of microbes, O_2_ dissolution and diffusion in soil water attenuate the temperature response of soil respiration. Overlooking these mechanisms in data analysis risks incorrectly ascribing their influence to biological factors, thereby overestimating the role of microbes and substrate heterogeneity in regulating the temperature response of soil respiration. The next generation of SOC models should therefore consider soil architecture and microscopic physical processes.

## Data Availability

Soil images used to calculate the results presented in this paper are available from https://figshare.com/articles/dataset/Soil_respiration/20198717.

## Appendix A

See table 1.

**Table 1.** Nomenclature.

*A_ws_*	specific water–pore wall interfacial area (cm²)
*A_wa_*	specific water–air interfacial area (cm²)
*A_w_*	specific water–pore wall interfacial area when soil is saturated (cm²)
*A_a_*	parameter in the specific water–air interfacial area (cm^−1^)
*C*_eq_	saturated dissolved O_2_ concentration at water–air interface (mg l^−1^)
*C_o_*	average dissolved O_2_ concentration at wetted pore wall (mg l^−1^)
*c_o_*	dissolved O_2_ concentration (mg l^−1^)
*c_D_*	dissolved organic carbon concentration (mg l^−1^)
*D*	diffusion coefficient of dissolved O_2_ (cm^2^ s^−1^)
*D_D_*	diffusion coefficient of dissolved organic carbon (cm^2^ s^−1^)
*E_a_*	activation energy (kJ mol^−1^)
*E_r_*	feedback factor
*k_D_*	Michaelis–Menten constant for dissolved organic carbon (mg l^−1^)
*k_o_*	Michaelis–Menten constant for dissolved O_2_ (mg l^−1^)
*L*	average distance between water–air interface and wetted pore wall (cm)
*N*	number of aerobic microbes in a unit volume of water
*n*	number of the aerobic microbes associated with a unit area of wetted pore wall
*R*	gas constant (J mol^−1^)
*R_h_*	heterotrophic respiration (μmol g^−1^ d^−1^)
*s_DOC_*	dissolution rate of polymerized carbon to a unit volume (mg l^−1^ s^−1^)
*T*	temperature (K)
*u*_max_	pro-exponential constant (mg s^−1^)
*v*_max_	maximum microbial consumption rate of O_2_ (mg s^−1^)
*Θ*	saturation
*α*	dissolution rate of gaseous O_2_ at water–air interface (cm s^−1^)
*μ*	parameter characterizing wetted pore wall areas
*τ*	parameter characterizing water–air areas
*σ*	parameter characterizing water–air areas
*λ*	thickness of the thin water layer inhabited by microbes (cm)
*κ*	constant control microbial uptake of O_2_ (l mg^−1^)
